# Fixing PSII: Membrane fluidity facilitates FtsH functions

**DOI:** 10.1093/plcell/koaf003

**Published:** 2025-01-07

**Authors:** Nora Flynn

**Affiliations:** Assistant Features Editor, The Plant Cell, American Society of Plant Biologists; Department of Botany and Plant Sciences, University of California Riverside, Riverside, CA 92507, USA

The thylakoid membrane of chloroplasts contains 2 photosystem complexes that initiate the conversion of light to usable energy during photosynthesis. However, the photosystems are vulnerable to oxidative damage in the highly reactive environment of thylakoid membranes. In particular, the core components of PSII, known as D1 and D2, frequently must be replaced to maintain activity.

The removal of inactive PSII core components is accomplished by the thylakoid-bound FILAMENTATION TEMPERATURE-SENSITIVE H (FtsH) complex, a heterohexamer consisting of FtsH1, FtsH2, FtsH5, and FtsH8 (Lindahl et al. 2000). To unfold and extract a substrate from the membrane, the FtsH subunits hydrolyze ATP using their ATPases Associated with diverse cellular Activities (AAA + ATPase) domains. Then, to degrade the substrate, the FtsH subunits each have protease domains (Janska et al. 2013). The efficiency of the FtsH complex to extract and degrade its targets, including D1, affects broader photosynthetic performance because there is a constant demand for revitalizing inactive PSII.

Under stressful conditions like heat or cold, there is an even greater need for continual turnover of damaged PSII core components. Temperature also affects the direct environment of membrane-bound proteins by altering membrane composition, which leads to more fluid membranes in hot conditions and more rigid membranes in cold (Los and Murata 2004). Could the physical state of the membrane influence proteostasis, specifically FtsH-mediated PSII repair? **Jingzhi Zhang and colleagues (Zhang et al. 2025)** dive into this question and explore how temperature-dependent alterations in membrane dynamics affect the functioning of the FtsH complex (see Figure).

**Figure.**
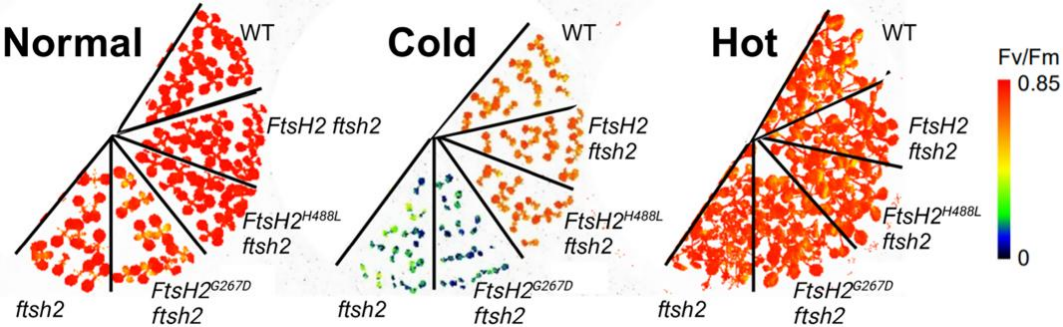
Temperature-dependent substrate extraction capabilities of the FtsH complex. Measurements of photochemical efficiency of the indicated Arabidopsis lines growing at 22 °C (normal), 10 °C (cold), or 30 °C (hot). Red indicates greater efficiency, while blue indicates lower efficiency. Adapted from Zhang et al. (2025), Figure 2. Created with BioRender.com.

The authors take advantage of Arabidopsis T-DNA knockout lines with defects in FtsH subunits to investigate the complex's performance in heat and cold. The loss of function of each of the unique FtsH subunits results in a range of phenotypes, with the loss of FtsH2 showing the most severe effects, including leaf variegation and lower photosynthetic efficiency compared with wild type (WT) under normal temperatures. Intriguingly, the photosynthetic defects of *ftsh2* (also known as *var2*) disappear with heat to phenocopy WT (see Figure). However, in cold conditions, the reverse is true, where both *ftsh2* and *ftsh5* (also known as *var1*) have more drastic reductions in photosynthetic efficiency compared with WT. This pattern suggests that greater membrane fluidity during heat could aid PSII repair by a compromised FtsH complex, allowing other FtsH subunits to compensate even when one subunit is nonfunctional.

What is causing the compromised FtsH complex to functionally collapse under cold but maintain activity in heat? The authors focus on disentangling the substrate extraction and proteolysis capabilities of the complex by using transgenic *ftsh2* lines that express FtsH2 variants with defects in either the AAA + ATPase or protease domains (see Figure). Under normal temperatures and cold, *ftsh2* expressing FtsH2^G267D^, which inactivates the AAA + ATPase domain and substrate extraction (but has an intact protease domain), demonstrates the same low photosynthetic efficiency as *ftsh2*. Conversely, *ftsh2* expressing FtsH2^H488L^, which inactivates the protease domain and substrate degradation (but has an intact AAA + ATPase domain), resembles WT (Zhang et al. 2010). This suggests that incorporating the AAA + ATPase domain's substrate extraction activity is sufficient to boost the PSII activity of *ftsh2* but providing protease activity is not. Again, under heat, the photosynthetic efficiency of all transgenic *ftsh2* lines was restored, providing further support that membrane fluidity impacts the efficiency of substrate extraction.

The temperature-dependent capabilities of FtsH-mediated PSII repair are also highlighted by tracking D1 and D2 turnover by immunoblot. In normal temperatures and in cold, *ftsh2* expressing FtsH2^H488L^ is sufficient to accomplish nearly WT-level turnover. Therefore, with more viscous membranes in cold, FtsH2's substrate extraction activity is indispensable. On the other hand, in heat, more fluid membranes could make substrate extraction easier, allowing the AAA + ATPase domains of the other FtsH subunits to fulfill D1 and D2 turnover requirements across *ftsh2* lines.

The impact of membrane fluidity on proteostasis is understudied but could uncover new pathways to engineer more thermoresistant plants and combat changing climate conditions. By revealing the temperature-dependent dynamics of the FtsH complex during PSII repair, the authors take a step toward a better understanding of how to protect photosynthetic productivity.

## Dive Curated Terms

The following phenotypic, genotypic, and functional terms are of significance to the work described in this paper:

var1 Gramene: AT5G42270
var1 Araport: AT5G42270

VAR2 Gramene: AT2G30950
VAR2 Araport: AT2G30950

FtsH2 Gramene: AT2G30950
FtsH2 Araport: AT2G30950

FtsH5 Gramene: AT5G42270
FtsH5 Araport: AT5G42270
